# Temperature Values Variability in Piezoelectric Implant Site Preparation: Differences between Cortical and Corticocancellous Bovine Bone

**DOI:** 10.1155/2016/6473680

**Published:** 2016-03-24

**Authors:** Luca Lamazza, Girolamo Garreffa, Domenica Laurito, Marco Lollobrigida, Luigi Palmieri, Alberto De Biase

**Affiliations:** ^1^Department of Oral and Maxillofacial Sciences, Sapienza University of Rome, 00161 Rome, Italy; ^2^Euro-Mediterranean Institute of Science and Technology, 90139 Palermo, Italy; ^3^Me. Di. Mediterranea Diagnostica, 80053 Castellammare di Stabia, Italy; ^4^National Centre of Epidemiology, Surveillance, and Promotion of Health, National Institute of Health, 00161 Rome, Italy

## Abstract

*Purpose*. Various parameters can influence temperature rise and detection during implant site preparation. The aim of this study is to investigate local temperature values in cortical and corticocancellous bovine bone during early stages of piezoelectric implant site preparation.* Materials and Methods*. 20 osteotomies were performed using a diamond tip (IM1s, Mectron Medical Technology, Carasco, Italy) on two different types of bovine bone samples, cortical and corticocancellous, respectively. A standardized protocol was designed to provide constant working conditions. Temperatures were measured in real time at a fixed position by a fiber optic thermometer.* Results*. Significantly higher drilling time (154.90 sec versus 99.00 sec; *p* < 0.0001) and temperatures (39.26°C versus 34.73°C; *p* = 0.043) were observed in the cortical group compared to the corticocancellous group. A remarkable variability of results characterized the corticocancellous blocks as compared to the blocks of pure cortical bone.* Conclusion*. Bone samples can influence heat generation during* in vitro* implant site preparation. When compared to cortical bone, corticocancellous samples present more variability in temperature values. Even controlling most experimental factors, the impact of bone samples still remains one of the main causes of temperature variability.

## 1. Introduction

Thermal trauma has been widely recognized as one potential cause of osteonecrosis following bone surgical procedures [1, 2]. In the specific field of dental implants, thermal injury has also been reported to cause early implant failure [3]. Most* in vitro* studies have thus addressed bone viability after thermal trauma [4]. Although different threshold values are reported in the literature [5–7], a temperature of 47°C for 1 minute is the most commonly accepted value to avoid bone injury [8]. In other words, thermal damage to bone is related to the magnitude of the temperature elevation and the duration of exposure.

Several factors contribute to temperature elevation during implant site preparation [9] but little is known about the specific contribution of each individually. Factors conducive to temperature elevation can be divided into three main groups: technique-, operator-, and bone-related factors. With respect to conventional drilling, technique-related factors include drill speed, cutting efficiency, and the cooling system. Applied load and motion pattern are to be related to operator. Despite efforts to standardize all the parameters involved during* in vitro *experiments, the anisotropic thermal behavior of bone introduces an additional factor that can have a major impact on temperature variation. Focusing on bone specimens, distinct features can thus be considered, that is, animal species, sample macrogeometry, cortical-medullary ratio, cortical thickness, bone mineral density (BMD), and thermal conductivity. Moreover, it is difficult to investigate the effect of one class of variables while maintaining constant other parameters.

More recently, use of piezoelectric devices has been proposed for implant site preparation [10, 11]. Selective and micrometric cut and bleeding control by cavitation effects are the main advantages of piezoelectric bone surgery [12, 13]. Histologic findings also showed good response of bone tissue after piezoelectric surgery compared to conventional techniques [14]. The use of piezoelectric technique for implant site preparation seems to positively affect osseointegration and implant stability [15] when compared to the traditional drilling technique. However, as in rotating techniques, a number of factors contribute to temperature elevation including technique-related factors (e.g., tip geometry and surface, internal or external irrigation), operator-related factors (applied load and motion pattern), and bone-related factors. Although piezoelectric technique has been shown to be reliable and effective, to date less data are available on local temperature rise, as the vast majority of studies published on the topic have been conducted using rotating techniques. Moreover, certain results variability has been observed in previous studies. The aim of this* in vitro* study has been to investigate local temperature values in cortical and corticocancellous bone samples during the early phases of piezoelectric implant site preparation. The primary question has been whether temperature variability could be related to bone specimens. The secondary question has been whether differences exist in temperature and osteotomy duration between the groups. A standardized protocol using a mechanical guiding device was adopted to control both technique and operating parameters.

## 2. Materials and Methods

### 2.1. Test Description

**Figure 1:**
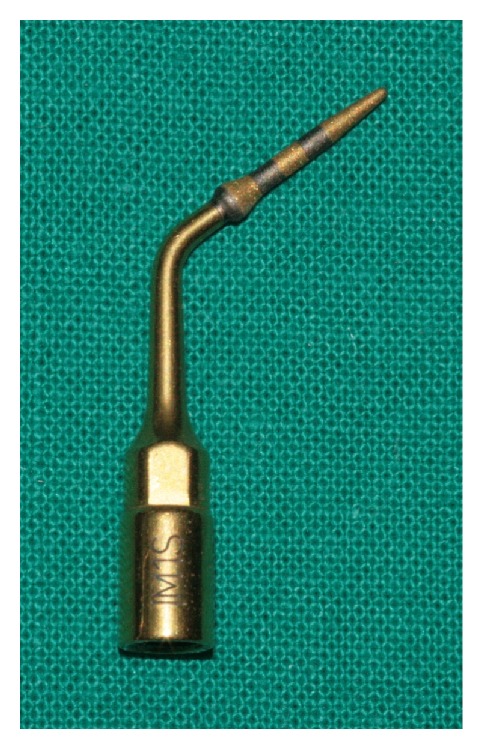
The diamond tip (IM1s, Mectron Medical Technology, Carasco, Italy) used in the tests. This tip can be regarded as a pilot drill in piezoelectric implant site preparation.

**Figure 2:**
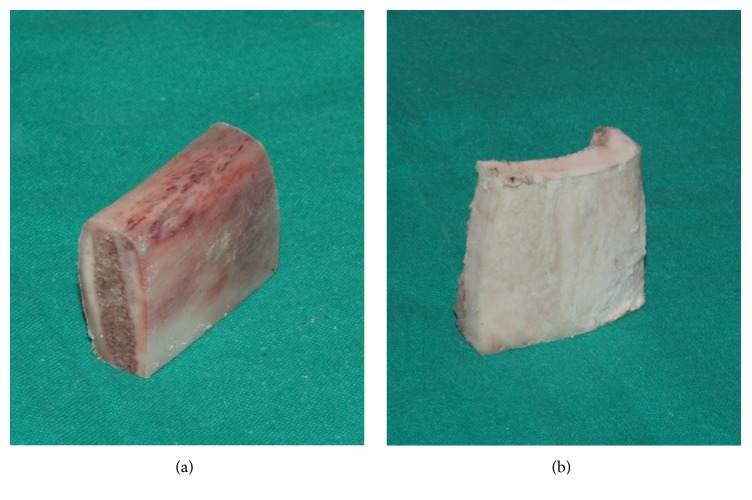
The two types of bone samples used in the tests. (a) Corticocancellous bone. (b) Cortical bone.

**Figure 3:**
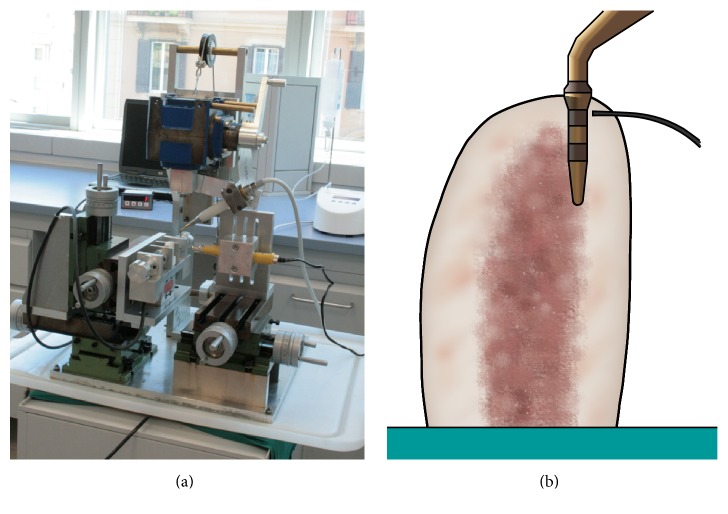
(a) The mechanical positioning device used in the study. By rotating the micrometric screws, a reproducible distance of 0.5 mm between the thermometer probe and the tip within the bone samples was obtained. Also visible, the drill (yellow-colored) used to prepare the holes for the thermometer probes. (b) Schematic section of a bone sample (corticocancellous) with the thermometer probe and the tip at completion of drilling.

A total of 20 osteotomies at a depth of 10 mm were performed using a diamond tip (IM1s, Mectron Medical Technology, Carasco, Italy) (Figure 1) in two different groups of bovine bone samples, cortical and corticocancellous, respectively. Corticocancellous specimens consisted of sectioned ribs of young bovine (Figure 2(a)), while cortical samples consisted of split shaft sections of the femur (Figure 2(b)). Both samples were collected from the same animals. A novel mechanical device was used to guarantee constant working conditions (Figure 3(a)). By the action of micrometer screws, bone samples and drill were moved in the three major axes in order to create the holes for the thermometer sensors. The piezoelectric handpiece was mounted on a transmission tool, equipped with handle, for both vertical and rotational manual movement. These movements were executed by a single expert operator. Temperatures were measured in real time and recorded using a fiber optic thermometer (Luxtron m 3300 Biomedical Lab Kit, Luxtron Corporation, Santa Clara, CA, United States). The detection point was first set at 0.5 mm from the tip surface, 8 mm from the tip head. Each bone sample was then moved so that the detection point was 2 mm below the top of the specimen (Figure 3(b)). Real time data were displayed on a screen using dedicated software (TrueTemp, LumaSense Technologies, Inc., Santa Clara, CA, United States). Three variables were considered for data analysis:

(i) *T*_max_ (°C): maximum temperature reached during the test;
(ii) *T*_60_ (°C): mean temperature on a 60 sec time interval around *T*_max_ (30 sec before and 30 sec after each *T*_max_ value);
(iii) duration (sec): number of seconds from the first tip-to-bone contact till a drilling depth of 9 mm was reached.

A load cell equipped with display showed the real time load applied on bone. The working load was maintained under 150 gr. Working cycles of 4 sec were adopted, as described in an earlier study [16]. Each cycle consisted of three different movements: longitudinal downward, rotational, and longitudinal upward. Bone samples were kept wet at all times, stored frozen in saline at −10°C, and used within 3 to 4 weeks. Osteotomies were performed at room temperature (24–26°C) with a baseline temperature of 20 ± 1.5°C.

### 2.2. Statistical Analysis

In order to investigate the temperature rise at our test point in the two different bone samples, mean, standard deviation, and median of the variables of duration, *T*_max_, and *T*_60_ were elaborated according to bone sample group. Correlation analysis was also performed on the variables. Given the number of samples, the Mann-Whitney nonparametric *U* test for independent samples was performed so as to compare the average duration, *T*_max_, and *T*_60_ between the two groups; the test of median was used for comparing medians; Levene's test using *F*-Fisher values was used for comparison of variances. Statistical significance was accepted at *p* = 0.05. The size of 10 osteotomies for each of the two samples assures a statistical power over 80% in the comparison of the mean values of duration and temperature between the two independent groups of bone samples under the hypothesis that variables were normally distributed, given the standard deviations and the differences calculated in each group and given the type 1 error probability of 0.05 associated with the null hypothesis that the population means of the two groups were equal.

## 3. Results

Tables 1 and 2 report the summary statistics for each variable of duration, *T*_max_, and *T*_60_ with nonparametric tests results for mean and median comparison.

**Figure 4:**
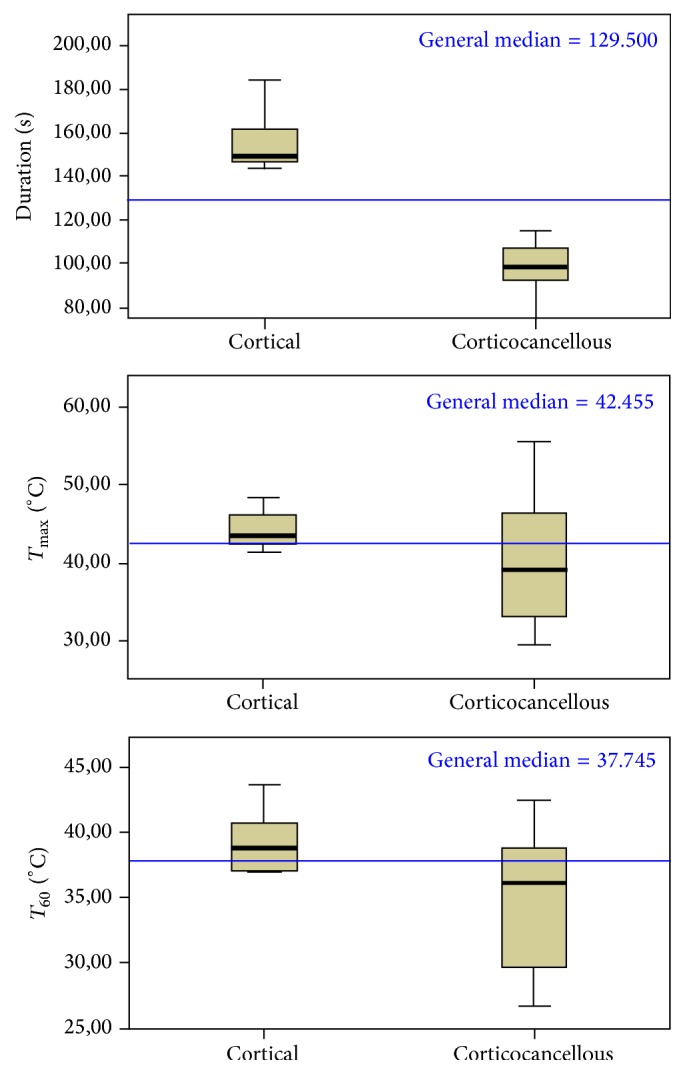
Box plots graphs showing the distribution of values for variables of duration, *T*_max_, and *T*_60_. Overall median values (continuous horizontal lines), specific median values by the two bone samples (continuous horizontal lines in the boxes), and ranges (whiskers) are shown. Statistically significant differences between the two bone samples median values were found for duration and *T*_60_ variables only.

Osteotomies had an average duration that was significantly higher (*p* < 0.0001) in cortical bone than in corticocancellous bone (154.90 sec versus 99.00 sec). Similar results were found for medians, with statistically significant (*p* < 0.0001) higher values for the cortical group (149.50 sec. versus 98.50 sec). Means and medians of *T*_max_ were higher in the cortical bone sample group than in the corticocancellous group (44.06 versus 40.07°C and 43.46 versus 39.04°C, resp.) but the differences were not statistically significant (*p* = 0.089 and *p* = 0.179, resp.). On the contrary, *T*_60_ values resulted in being significantly higher in the cortical bone group (39.26°C versus 34.73°C; *p* = 0.043); median values proved higher in the cortical bone sample group than in the corticocancellous samples, but these differences were not statistically significant. A graphical representation of the data is provided in Figure 4.

As reflected by standard deviations, the temperature values resulted in being less dispersed in the cortical group: the dispersion of *T*_max_ values was about three times higher in the corticocancellous group than in the cortical group (8.0 versus 2.4, resp.). As for *T*_60_, standard deviation was about double (5.2 versus 2.3, resp.). In addition, the *F*-Fisher test for comparison of variances was statistically significant for both *T*_max_ (*F* = 6.484; *p* = 0.020) and *T*_60_ (*F* = 9.663; *p* = 0.006) confirming a different variability in the two groups (Table 3).

Test duration presented a positive correlation with *T*_max_ (*ρ* = 0.404), even if not statistically significant (*p* = 0.077), and with *T*_60_ (*ρ* = 0.604; *p* = 0.005) (data analysis not reported).

## 4. Discussion

Bone necrosis related to high temperatures is a well-known phenomenon observed in differing surgical specialties [17]. Up to now, most of the research concerning heat generation during bone surgery has involved* in vitro* studies [18]. A number of methods have been developed to investigate various techniques of bone instrumentation, for example, traditional drilling, ultrasound, and laser devices. However, since different factors play specific roles in each technique, a comprehensive and unique approach to the study of heat generation in bone tissue has not been developed as yet [19]. Furthermore, the use of bone samples from different animal species makes it difficult to compare studies generating different results. In the present study, by using a mechanical positioning device, the technical and operator-related factors were controlled, thus focusing the analysis on bone thermal response.

**Figure 5:**
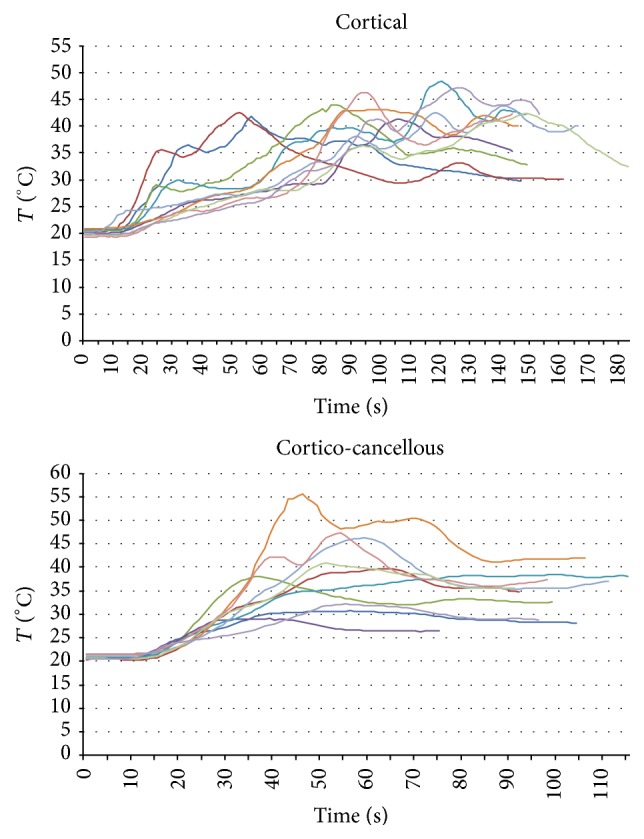
Comprehensive graphs showing temperature trends in cortical and corticocancellous bone samples.

From our results, no statistically significant differences were found for *T*_max_ values between the groups, while mean temperature and osteotomy duration resulted in being significantly higher in the cortical group. Remarkably, different results were obtained depending on temperature parameters (*T*_max_ and *T*_60_), with lower values and statistically significant difference between the groups only for the *T*_60_ values. As it is shown in Figure 5, temperature values present different trends in the two groups; peaks too are differently scattered along the timeline. Moreover, temperature peaks show different shapes, indicating different relevance. In fact, a high but abrupt temperature rise in the context of an overall low thermal response may have less relevance than a moderate but protracted temperature rise. This suggests that *T*_max_ alone cannot be regarded as a reliable indicator of bone thermal response in this kind of test, in which temperature is affected by specific bone characteristics. By contrast, mean temperature around the maximum may be more representative of overall bone thermal response. As evidenced in the graphs cited above, bone thermal response would seem to be less smooth in the cortical group.

Apparent disorder in the cortical group is, however, a result of peak distribution along the timeline. Conversely, the wider vertical distribution of temperature peaks in the corticocancellous group reveals the variability of thermal behavior as reflected by the analysis of variance. These findings suggest that heterogeneous bone samples are characterized by a wider variability in measured temperature values during ultrasonic implant site preparation.

Variable results may therefore be related to specimens and to experimental errors [20], despite the great effort expended in this study to minimize the latter. If, on the one hand, it is proven that conventional drilling in cortical bone produces higher temperature than in cancellous bone [21, 22], less is known about the intrinsic thermal behavior of different kinds of bone specimens. Bone is commonly considered as thermally anisotropic [23–25]. However, Davidson and James [26] concluded that bovine cortical bone can be considered as thermally isotropic. Regardless of differences in test conditions, it can be argued that the variability of results found in the corticocancellous group derives from differences in cortical thickness and from the structural complexity of cancellous bone (mineral composition, fluid dynamics, trabecular orientation, etc.).

From a microscopic viewpoint, BMD seems an important feature in determining bone thermal response. Karaca et al. [27] reported a positive correlation between BMD and temperature rise using samples from bovine tibia. The authors specify that temperatures were recorded at a distance of 0.5 mm from the hole drilled but did not clarify the probe's exact location (cortical or medullary). Although BMD is expected to be higher in the cortical layer, bone hardness can differ from one sample to another, as well as in different sites of the same specimen. Clearly, the greater the cortical thickness, the greater the “cortical” effects on temperature rise. Sener et al. [28] reported higher temperatures in cortical bone than in cancellous bone with conventional drilling techniques. However, only one type of bone samples (fresh bovine mandibles) was used in this study, basing the distinction between cortical and cancellous bone on the depth of the probe. Similarly, Rashad et al. [29] found higher temperatures in cortical bone during ultrasonic preparation. Yet these results are not directly comparable with other studies since data from multiple tips were pooled. Stelzle et al. [30] compared piezoelectric implant site preparation with conventional drilling using pig calvaria, which consisted of thin cortical layer and dense cancellous bone. Piezoelectric technique showed the highest mean temperatures, positively correlated with longer osteotomies durations. Compared to our results, the temperatures recorded in this study are, on the whole, lower (38.0 ± 2.7 with 100–200 g load applied). This is likely to be related to the different tip (IM3, Mectron Medical Technology) and cooling system utilized. Moreover, the 2 mm distance between the thermal probe and the implant site may have discarded sudden temperature variations.

In our study, a significantly longer drilling time was observed in the cortical samples. Higher mean temperatures observed in the cortical samples may therefore be the overall result of compact bone resistance, prolonged drilling time, and hence enhanced heat production due to frictional forces. In order to comprehend bone thermal behavior, its structural and mechanical properties should also be taken into account. Bone can be described as a composite material made of different structures, hierarchically organized on different dimensional scales [31]. From this perspective, the distinction between cortical and trabecular bone takes into account only the macrostructural level. Nevertheless, even considering pure cortical bone, heterogeneity can arise from variable microstructural parameters such as porosity and percentage mineralization [32]. Thus, for instance, in analyzing the mechanical properties of compact bone, Currey [33] demonstrated a strong positive relationship between Young's modulus and both calcium content and volume fraction. It is likely that such heterogeneity will be reflected in thermal behavior, with more evident effects in trabecular bone where substantial variations are apparent even at the macroscopic level. As suggested by Davidson and James [26], thermal properties such as conductivity and heat capacity not only depend on the intrinsic properties of the material itself, but are also influenced by its structural organization. A comprehensive study of the interrelationship between mechanical and thermal properties of bone has however yet to be conducted.

As a result, some authors propose use of artificial bone specimens to overcome the limitations imposed by traditional* ex vivo* bone samples [34, 35]. According to these authors, synthetic bone specimens provide homogeneous characteristics together with thermal conductivity similar to that of human bone.

It is interesting finally to note that the influence of bone sample characteristics has rarely been considered of pivotal importance when dealing with the rotating technique [36]. This derives from the different “sensitivity” of rotating and ultrasonic techniques with respect to bone features. The mechanical energy applied in rotating techniques is much greater than the mechanical resistance of the finest bone characteristics, so that their influence on temperature may be ignored by a measuring apparatus, whereas ultrasonic vibrations provide a “gentler” action, involving less local delivery of mechanical energy. As a result, even the finest bone structures possess nonnegligible effects in terms of heat generation and measurement.

This preliminary study has certain limitations. First, even though the osteotomies were performed by a single expert operator with a real time pressure displaying device, this does not represent complete standardization of working conditions. In addition, recording and analysis pressure data during bone drilling might reveal more precise correlations between applied load and temperature rise.

## 5. Conclusion

Temperature rise during bone drilling is a very complex phenomenon affected by many variables. Although some operator-related variables can be minimized (though not totally eliminated), less can be done to exclude variabilities resulting from bone samples. Pure cortical bone samples were characterized by lower temperature variability during* in vitro* tests; however they do not represent actual clinical conditions. Differently, corticocancellous samples are a better simulation of* in vivo* conditions but were affected by greater variability of results. Cortical samples also showed longer osteotomies duration and higher mean temperatures. Given the difficulty in controlling some of the bone-related variables that are likely to be important factors in heat generation, the lack of standardization of technique- and operator-related factors may lead to increased variability in temperatures and risk of overheating.

## Figures and Tables

**Table 1:** Means, standard deviations, and comparison of means using Mann-Whitney *U* test for duration, *T*_max_, and *T*_60_, in the two bone samples.

	Cortical		Corticocancellous		Mann-Whitney *U* test
	Mean	SD	Mean	SD	*p* value
Duration (sec)	154.90	12.7	99.00	11.7	<0.0001
*T*_max_ (°C)	44.06	2.4	40.07	8.0	0.089
*T*_60_ (°C)	39.26	2.3	34.73	5.2	0.043

**Table 2:** Medians and comparison of medians using the test of median for duration, *T*_max_, and *T*_60_ in the two bone samples.

	Cortical	Corticocancellous	Test of median
	Median	Median	*p* value
Duration (sec)	149.50	98.50	<0.0001
*T*_max_ (°C)	43.46	39.04	0.179
*T*_60_ (°C)	38.85	36.11	0.656

**Table 3:** *F*-Fisher values and statistical significance (*p* value) for variance comparison between the two bone samples using Levene's test for duration, *T*_max_, and *T*_60_.

	*F* ^*∗*^	Sig.
Duration (sec)	0.10	0.75
*T*_max_ (°C)	6.48	0.02
*T*_60_ (°C)	9.66	0.006

^*∗*^Equal variances assumed.
